# Low high-density lipoprotein cholesterol levels are associated with malignant intraductal papillary mucinous neoplasms: A multicenter study

**DOI:** 10.1186/s12944-021-01523-8

**Published:** 2021-08-28

**Authors:** Cheng Wang, Tingting Lin, Xinru Wang, Zhicheng Yu, Xiaoling Zhuge, Wenjing Cui, Miaomiao Wang, Zhongqiu Wang, Chuangen Guo, Xiao Chen

**Affiliations:** 1 Department of Radiology, Nanjing Drum Tower Hospital, The Affiliated Hospital of Nanjing University Medical School, 321 Zhongshan road, 210008 Nanjing, China; 2 Department of Radiology, the Affiliated Hospital of Nanjing University of Chinese Medicine, 155 Hanzhong road, 210029 Nanjing, China; 3 Department of Laboratory Medicine, the First Affiliated Hospital, College of Medicine, Zhejiang University, 79 Qingchun road, 310003 Hangzhou, China; 4 Department of Radiology, the First Affiliated Hospital, College of Medicine, Zhejiang University, 79 Qingchun road, 310003 Hangzhou, China

**Keywords:** lipids, HDL-cholesterol, intraductal papillary mucinous neoplasms, pancreas, malignancy, invasive carcinoma, branch duct

## Abstract

**Background:**

Intraductal papillary mucinous neoplasms (IPMNs) can potentially undergo malignant transformation. Studies have shown that high-density lipoprotein cholesterol (HDL-c) was associated with the risk of cancer. In this study, the association between HDL-c and the incidence of malignancy in IPMNs was investigated.

**Materials and methods:**

226 patients with histologically proven IPMNs who underwent surgery were included in the present study. Patients were assigned to a training group (*n* = 151) and validation group (*n* = 75). Patients’ demographic information, clinical data, and histopathological evaluation findings were obtained from medical records. Malignant IPMNs were defined as lesions that showed high grade dysplasia and invasive carcinoma. Logistic regression analyses were used to show the association between HDL-c and malignant IPMNs. Receiver operating characteristic (ROC) curves were generated to analyze predictive performance.

**Results:**

The prevalence of low HDL-c levels was higher in patients with malignant IPMNs than in those with non-malignant IPMNs (*P* < 0.01) in both the training group and validation group. The prevalence of malignant IPMNs decreased with an increase in HDL-c levels both in patients with all types of IPMNs, as well as in those with branch-duct IPMNs (BD-IPMNs).Logistic analysis showed that low HDL-c levels were associated with malignant IPMNs (odds ratio (OR) = 20.56, 95 % confidence interval (CI): 2.58–163.64, *P < 0.01*) in all types of IPMNs and BD-IPMNs (OR = 17.6, 95 %CI: 1.16–268.46, *P = 0.02* ).The predictive performance of mural nodules plus low HDL-c levels was higher than that of mural nodules alone or mural nodules plus cyst size for the identification of malignant BD-IPMNs.

**Conclusions:**

HDL-c levels may serve a potential biomarker for identifying malignant IPMNs and improve the predictive ability of malignancy in BD-IPMNs.

## Introduction

Intraductal papillary mucinous neoplasms (IPMNs) are common cystic neoplasms of the pancreas. IPMNs can potentially undergo malignant transformation, with the development of invasive carcinoma in some patients [1]. Histopathologically, IPMNs have the potential to progress from low-grade dysplasia to invasive carcinoma over the time [2, 3]. The incidence of high-grade dysplasia or pancreatic cancer in IPMNs is 42 % [4]. IPMNs are categorized into the following types based on the degree of involvement of the pancreatic ductal system: main duct (MD), branch duct (BD), and mixed type IPMNs (showing features of MD and BD). In view of the high risk of malignancy, surgical intervention is recommended for MD-IPMNs and mixed IPMNs with main pancreatic duct (MPD) > 10 mm, jaundice or mural nodules [5].Management of BD-IPMNs remains challenging.

High-risk stigmata and worrisome features include the main pancreatic duct (MPD) diameter and an enhanced mural nodule > 5 mm that are used as predictors of high-grade dysplasia or invasive carcinoma [6]. Several studies have also shown that the elevated tumor biomarkers including carcinoembryonic antigen (CEA) and serum carbohydrate antigen 19 − 9 (CA19-9) levels are associated with invasive carcinoma in IPMNs [5]. However, the predictive ability of these biomarkers remains unsatisfactory because of their inadequate sensitivity (lower than 50 %) and specificity [6]. There are inconsistencies among published guidelines [7]. Cut-off value of cyst diameter is set at 4.0 cm in European guideline [8], but it is set as 3.0 cm in Fukuoka guideline [6].The follow-up management is also different among guidelines. In addition, a few studies have reported the use of an extracellular vesicle as a novel approach to predict malignant IPMNs [9]. However, few biomarkers have been reported to identify malignancy in BD-IPMNs.

Cholesterol plays a critical role in cancer progression by enhancing cell proliferation, migration, and invasion [10]. High-density lipoprotein (HDL) is the predominant lipoprotein cholesterol that transports cholesterol into major steroidogenic organs [11, 12]. The anticancer action of HDL is attributed to its pleiotropic properties, including its anti-oxidative action, modulation of cytokine production, inhibition of apoptosis, as well as promotion of cell growth and migration [13, 14]. HDL potentially antagonizes the two primary hallmarks of cancer progression via its potent antioxidant and anti-inflammatory activity [15, 16]. The association between high density lipoprotein-cholesterol (HDL-c) and cancer risk is also reported, including breast cancer [12, 17], prostate and ovarian cancer [12]. A similar correlation was also observed between HDL-c levels and risk of gastrointestinal cancer, such as liver cancer [18, 19], biliary tract cancer [20, 21] and pancreatic cancer [12, 22, 23]. However, to our knowledge, no study has investigated the association between HDL-c levels and IPMNs. In this multicenter study the association between HDL-c levels and the incidence of malignancy in IPMNs, particularly in BD-IPMNs, was investigated.

## Materials and methods

### Patients

This multicenter (three-center) study included 226 patients with biopsy-proven IPMNs who underwent surgery during 2011–2020 after excluding those patients with data missing or receiving chemotherapy/radiotherapy. Patients were categorized into the following groups: 151 patients in center 1 and center 2 were set as training group and 75 patients in center 3 was assigned to validation group. Patients’ demographic information, clinical data, and pathological features were obtained from medical records. The following data: preoperative symptoms (such as abdominal symptoms and overt jaundice), serum carbohydrate antigen 19 − 9 (CA19-9) level, serum carcinoembryonic antigen (CEA) levels, HDL-c, low-density lipoprotein (LDL), triglyceride (TG) and total cholesterol (TC) levels, were recorded. Clinical biochemical test was performed by the automatic biochemical analyzer (210 and 7480 Hitachi, Japan). The medical history of pancreatitis and diabetes mellitus (DM) was also collected. A cut-off value of 0.7 mmol/L was chosen for HDL-c levels based on previous studies [19, 22]. Fasting and 2-hour plasma glucose levels were determined within one week preoperatively. DM was defined according to plasma glucose levels and a history of DM. Imaging information was obtained from the picture archiving and communication system. This retrospective study was approved by the Institutional Ethics Review Board of the First Affiliated Hospital, Zhejiang University School of Medicine. Informed consent was waived.

### Imaging analysis

All patients underwent magnetic resonance imaging (MRI) examinations (Signa HDx 3.0T, GE Medical Systems, USA; or Achieva 3.0T, Philips, The Netherland). T1 weighted imaging (T1WI), T2WI, magnetic resonance cholangiopancreatography and contrast-enhanced imaging were obtained. The following imaging findings were recorded: MPD diameter, enhanced mural nodules, cyst location, cyst size and the communication with the MPD, if any.

### Malignant intraductal papillary mucinous neoplasms

IPMN was classified into three types based on the extent of involvement of the pancreatic ductal system: the MD, BD, and mixed types. Histopathologically, IPMNs was categorized as low-intermediate dysplasia, high-grade dysplasia, or invasive adenocarcinoma. Malignant IPMNs were defined as those which showed features of high-grade dysplasia and associated invasive carcinoma.

### Statistical analysis

Based on HDL-c levels, patients were categorized into the following groups: those with HDL-c levels < 0.7 mmol/L and those with HDL-c levels ≥ 0.7 mmol/L. The two-tailed t tests or Mann-Whitney U-test was used to compare the variables. Univariate and multivariate logistic regression analyses were used to determine the association between low HDL-c levels and malignant IPMNs. The results were adjusted for diabetes and pancreatitis. Receiver operating characteristic (ROC) curves were generated to analyze the performance of mural nodules plus low HDL-c levels as predictor of malignant IPMNs. A p value less than 0.05 was considered statistically significant.

## Results

### Patients’ clinical data

**Fig. 1:**
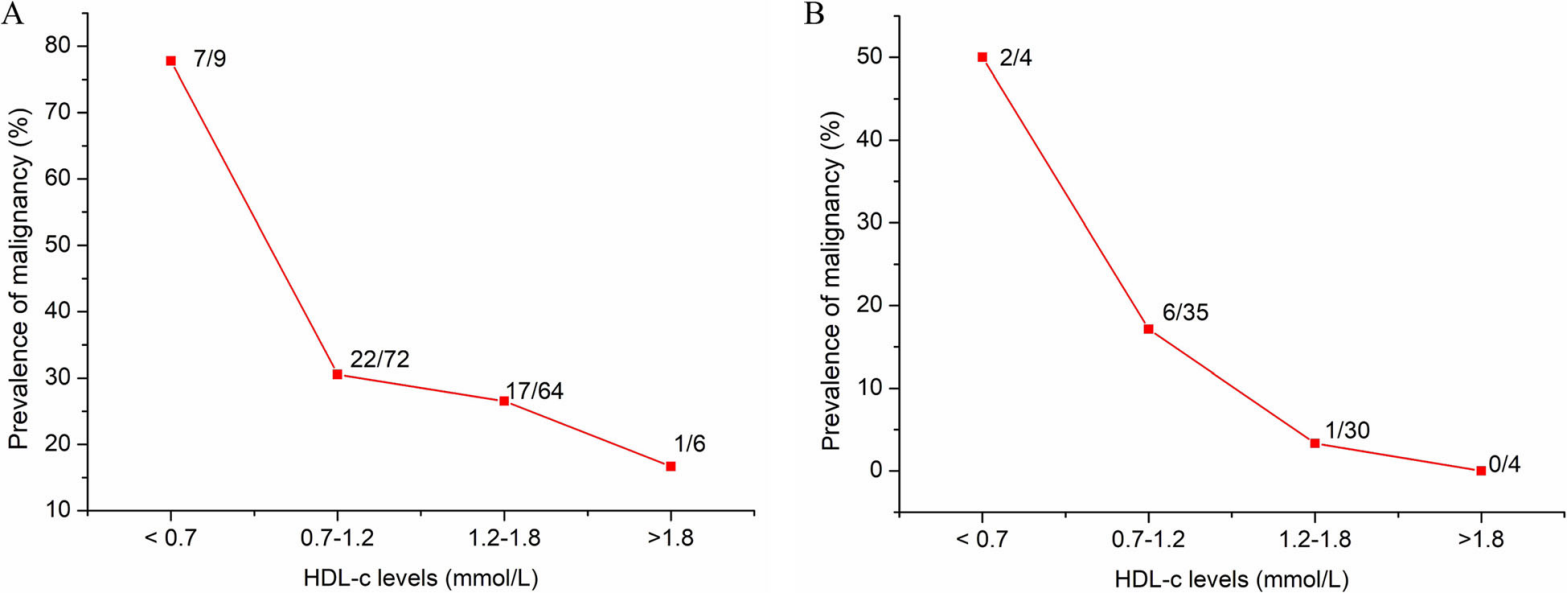
The association between the prevalence of malignant intraductal papillary mucinous neoplasms (IPMNs) and high-density lipoprotein cholesterol (HDL-c) levels. The prevalence of malignancy decreased with an increase of HDL-c in all IPMNs (**A**) and branch duct intraductal papillary mucinous neoplasm (BD-IPMNs) (**B**)

The clinical data of patients in training group and validation group is shown in Table 1. Malignant IPMNs were diagnosed in 47 patients (31.1 %) in the training group and in 19 patients (25.3 %) in the validation group. The prevalence of low HDL-c levels was higher in patients with malignant IPMNs than in patients with non-malignant IPMNs (*P* < 0.01) in both training and validation groups. Of the patients with low HDL-c levels, 77.8 % had malignant IPMNs. Similar result was observed in validation group (75.0 %). The prevalence of malignant IPMNs tended to decrease with an increase in HDL-c levels in patients with all type IPMNs and BD-IPMNs (Fig. 1).

**Table 1:** Clinical data in malignant and non-malignant IPMNs

	Training group (center 1 and Center 2)				Validation group (center 3)			
	Total (*n* = 151)	Malignant IPMN (*n* = 47)	Non-malignant IPMN (*n* = 104)	*P*	Total (*n* = 75)	Malignant IPMN (*n* = 19)	Non-malignant IPMN (*n* = 56)	*P*
Age	63.28 ± 9.47	63.59 ± 8.99	62.60 ± 10.50	0.55	64.8 ± 8.90	68.42 ± 8.25	63.68 ± 8.86	0.44
Size	3.69 ± 1.98	3.93 ± 1.63	3.58 ± 2.11	0.34	3.1 ± 1.12	3.8 ± 1.33	2.5 ± 0.93	0.02
Sex(male/female)	95/56	34/13	61/43	0.11	49/26	11/8	38/18	0.43
Dysplasia				/				/
Low-intermediate grade	104	/	104		56	0	56	
High-grade	24	24	0		11	11	0	
Invasion	23	23	0		8	8	0	
Type				< 0.01				< 0.01
Main	24	16	8		14	8	6	
Branch	73	9	64		46	6	40	
Mixed	54	22	32		15	5	10	
Location				0.11				0.94
Head-neck	95	34	61		41	9	32	
Body and Tail	56	13	43		34	10	24	
CEA (ng/ml)	3.57 ± 3.61	4.75 ± 5.65	3.03 ± 1.95	0.09	3.99 ± 10.03	8.51 ± 19.29	2.43 ± 1.59	0.03
CA19-9 (U/ml)	48.68 ± 228.65	99.04 ± 396.08	26.19 ± 70.67	0.002	71.37 ± 204.27	132.83 ± 270.4	50.15 ± 174.53	0.13
HDL-c (mmol/L)	1.19 ± 0.39	1.10 ± 0.52	1.21 ± 0.31	0.38	1.02 ± 0.39	0.84 ± 0.41	1.08 ± 0.37	0.02
HDL-c < 0.7	9	7	2	< 0.01	12	9	3	< 0.01
LDL (mmol/L)	2.51 ± 0.82	2.52 ± 0.69	2.51 ± 0.89	0.95	2.27 ± 0.69	1.91 ± 0.65	2.40 ± 0.67	0.01
MPD diameter	0.61 ± 0.41	0.90 ± 0.49	0.48 ± 0.29	< 0.001		0.97 ± 0.42	0.43 ± 0.21	< 0.01
Pancreatitis	3	0	3	0.24	1	0	1	0.25
Diabetes	23	9	14	0.13	4	1	3	1.0
Lymph node metastasis (yes vs. no)	2	2	0	0.09		0	0	/
Peripancreatic extension	5	5	0	0.003	1	1	0	0.45
Mural nodule	17	13	4	< 0.01	8	5	3	0.01

Malignant intraductal papillary mucinous neoplasms (IPMNs) were defined as those with high grade dysplasia and associated invasive carcinoma

CA 19 − 9: carbohydrate antigen 19 − 9; CEA: carcinoembryonic antigen; MPD: main pancreatic duct; HDL-c: high-density lipoprotein-cholesterol; LDL: low density lipoprotein

### The association between low HDL-c and malignant IPMNs

Univariate and multivariate logistic regression analyses were used to identify the associated factors for malignant IPMNs (Table 2). Compared with MD involved IPMNs, BD-IPMNs had a lower risk for malignancy (OR = 0.13, 95 % CI: 0.05–0.34). However, this association was no longer present after adjustment for high risk stigmata (diameter of MPD and mural nodules). Low HDL-c levels were associated with the presence of malignant IPMNs (OR = 8.93, 95 % CI: 1.78–44.80), and such association remained after adjustment for DM, pancreatitis and serum LDL levels (OR = 20.56, 95 % CI: 2.58–163.64). A similar trend was observed in the validation group (OR = 6.66, 95 %CI: 1.08–41.06).

**Table 2:** Associated factors with malignant IPMNs

	Training group (center 1 and center 2)						Validation group (center 3)			
variables	Univariable		Multivariable				Multivariable			
	OR (95 %CI)	*P*	Model 1	*P*	Model 2	*P*	Model 1	*P*	Model 2	*P*
			OR (95 %CI)		OR (95 %CI)		OR (95 %CI)		OR (95 %CI)	
Mural nodule (yes vs. no )	9.56 (2.92–31.30)	< 0.01	15.14 (3.10–74.07)	< 0.01	14.51 (3.05–69.02)	< 0.01	16.11 (2.78–93.5)	< 0.01	25.2 (3.43–185.4)	< 0.01
Duct (cm)	18.0 (5.84–55.52)	< 0.01	10.45 (1.78–61.17)	0.02	11.61 (1.88–71.65)	0.02	15.4 (2.36–89.6)	< 0.01	18.76 (3.32–165.3)	< 0.01
Size (cm)	1.09 (0.91–1.30)	0.42	0.99 (0.79–1.23)	0.97	0.95 (0.76–1.19)	0.71	1.07 (0.86–1.28)	0.68	1.06 (0.86–1.31)	0.70
Type		< 0.01		0.11		0.10		< 0.01		< 0.01
MD + MT	1		1		1		1		1	
Branch-duct	0.15 (0.07–0.34)		0.39 (0.10–1.53)		0.38 (0.09–1.53)		0.21 (0.10–0.37)		0.16 (0.09–0.28)	
Location		0.11		0.28		0.38		0.49		0.53
Head-neck	1		1		1		1		1	
Body-Tail	0.54 (0.26–1.15)		0.57 (0.22–1.50)		0.62 (0.23–1.63)		0.47 (0.18–1.21)		0.50 (0.20–1.27)	
HDL-c (< 0.7 vs. ≥ 0.7 mmol/L)	8.93 (1.78–44.80)	< 0.01	17.92 (2.40–134.17)	< 0.01	20.56 (2.58–163.64)	< 0.01	6.63 (1.14–38.47)	0.02	6.66 (1.08–41.06)	0.02

Model 1 was adjusted with body mass index, age and gender; Model 2 was additionally adjusted with pancreatitis, low density lipoprotein levels and diabetes

HDL-c: high-density lipoprotein cholesterol; MD: main duct; MT: mixed type

Subsequently, the association between low HDL-c levels and malignancy in BD-IPMNs was observed (Table 3). Subgroup analysis also showed that low HDL-c levels were associated with the presence of malignant IPMNs (OR = 17.6, 95 % CI: 1.16–268.46) in BD-IPMNs.

**Table 3:** Associated factors with malignant IPMNs in BD-IPMN

variables	Univariable		Multivariable			
	OR (95 %CI)	*P*	Model 1	*P*	Model 2	*P*
			OR (95 %CI)		OR (95 %CI)	
Mural nodule (yes vs. no)	24.80 (3.61–170.3)	< 0.01	50.6 (5.12–499.6)	< 0.01	52.8 (5.29–528.2)	< 0.01
Duct (cm)	1.24 (0.01–170.5)	0.93	19.45 (0.04–1000.0)	0.35	17.6 (0.04–820.7)	0.35
Size (cm)	1.12 (0.86–1.46)	0.40	1.08 (0.77–1.52)	0.65	1.11 (0.78–1.56)	0.95
HDL-c (< 0.7 vs. ≥ 0.7 mmol/L)	8.86 (1.07–73.1)	0.04	15.27 (1.19-196.26)	0.04	17.6 (1.16-268.46)	0.02

Model 1 was adjusted with BMI, age and gender; Model 2 was additionally adjusted with pancreatitis, low density lipoprotein levels and diabetes

CI: confidence interval; HDL-c: high-density lipoprotein cholesterol; OR: odds ratio

### ROC analysis

**Fig. 2:**
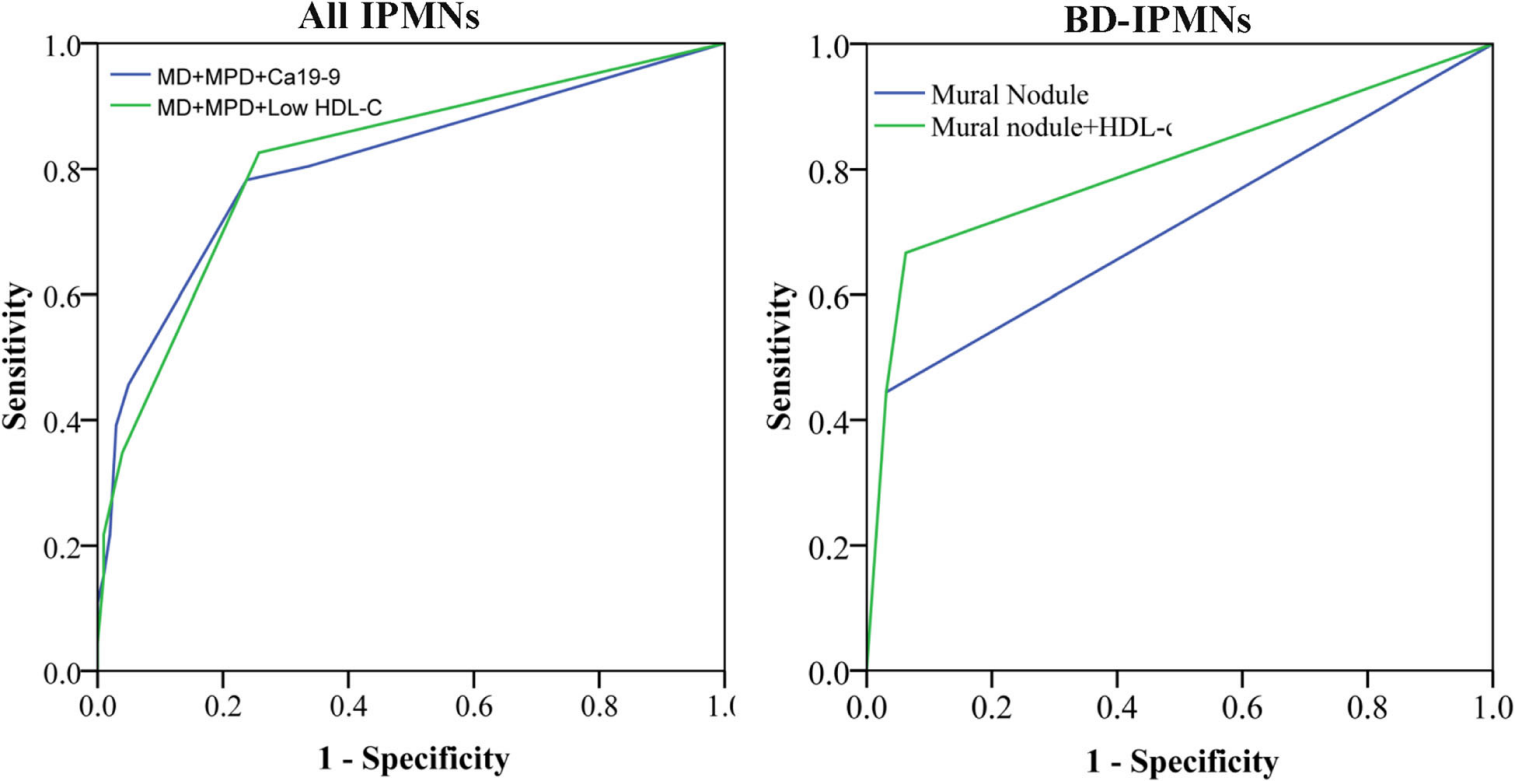
The receiver operating characteristic (ROC) curves of variables in predicting malignant intraductal papillary mucinous neoplasms (IPMNs). **A**: mural nodule plus MPD diameter and Ca19-9 or low high-density lipoprotein cholesterol (HDL-c) levels in predicting malignant IPMNs. **B**: mural nodule alone, mural nodule plus cyst size, and mural nodule plus low HDL-c levels in predicting malignant branch duct IPMNs (BD-IPMNs)

The ROC curve showed that the area under the curve (AUC) of mural nodules plus the MPD diameter and low HDL-c levels (AUC = 0.82, 95 % CI: 0.74–0.89, *P* < 0.01) was slightly higher than that of mural nodule plus MPD diameter and Ca19-9 levels (AUC = 0.81, 95 % CI: 0.73–0.89, *P* < 0.01) in identifying malignant IPMNs (Fig. 2). Moreover, the area under the curve (AUC) of mural nodules plus low HDL-c (AUC = 0.81, 95 % CI: 0.62–0.99, *P* < 0.01) was higher than that of mural nodules alone (AUC = 0.71, 95 % CI: 0.49–0.92, *P* = 0.046) or mural nodules plus the cyst size (AUC = 0.76, 95 %CI: 0.57–0.95, *P* = 0.02) for prediction of malignant BD-IPMNs (Fig. 2).

## Discussion

The diagnosis and clinical management strategies of patients with IPMNs remain controversial currently owing to the lack of accurate predictors of malignant transformation in these lesions [24, 25]. Tumor biomarkers, such as CEA and CA19-9levels have been used to predict the malignancy [5]. This study showed an inverse association between HDL-c levels and IPMNs. The prevalence of low HDL-c levels was significantly higher in the malignant IPMN than in the non-malignant IPMN group. Multivariable logistic analysis further showed that low HDL-c levels were associated with occurrence of malignant IPMNs. In addition, the combination of mural nodules and low HDL-c levels showed higher performance than that of mural nodules alone or of a combination of mural nodule and cyst size in predicting malignant BD-IPMNs. Low HDL-c levels may be potential biomarkers for malignant IPMNs.

Studies have reported an inverse relationship between serum TC levels and cancer risk [26–28]. Studies have also reported an association between HDL-c levels and cancer risk, as well as disease free survival and overall survival in many type of malignancies [12, 17–23, 29]. Low HDL-c levels were associated with a high TNM stage and occurrence of distant metastasis [30]. Notably, several studies have reported an inverse association between HDL-c levels and gastrointestinal cancer. Randomized controlled intervention studies have reported [22] that the inverse associations between HDL-c levels and pancreatic cancer was not materially affected by other lipids, and the inverse association remained unchanged after exclusion of the first 3 years of follow-up. Michalaki et al. [23] reported that serum HDL concentration was significantly lower in patients with pancreatic cancer than in the controls. Compared with the control group, patients with pancreatic cancer showed at least 2-fold lower serum apolipoprotein A-II, transthyretin, and apolipoprotein A-I levels [31]. HDL-c levels were inversely associated with the risk of liver cancer [18], and preoperative HDL-c levels were associated with the risk of cancer recurrence [19]. Compared with the reference group, patients in the lowest quintile of HDL-c (< 30 mg/dL) had an 11.6-fold higher risk of gallbladder cancer and a 16.8-fold higher risk of bile duct cancer [21]. Moreover, raising HDL levels may be a potential therapeutic strategy for against cancer [32]. However, the association between HDL-c levels and malignant IPMNs has not been clarified.

Interestingly, this study showed that a low serum HDL-c level was an independent risk factor for malignancy in IPMNs both in the training and validation groups even after adjustment for confounders. In addition, the diagnostic performance of mural nodules plus the MPD diameter and low HDL-c was similar to that of mural nodules plus the MPD diameter and CA19-9 levels. Management of BD-IPMNs remains challenging [33]. Few biomarkers are available for the diagnosis of malignant transformation in BD-IPMNs. Our data showed that low serum HDL-c levels improved the diagnostic performance of mural nodules in predicting malignant BD-IPMNs. The AUC increased from 0.71 to 0.81. Interestingly, we also observed an inverse association between low HDL-c levels (< 0.8 mmol/L) and grade 3 pancreatic neuroendocrine neoplasms (PNENs) (*n* = 197, data not shown). These data further support the finding that low HDL-c levels are associated with pancreatic malignancy. However, conflicting evidence was also reported between HDL levels and cancer incidence or mortality because this association may depend upon tumor type [32]. However, an increasing body of evidence supports the association [12, 32]. The association between HDL and IPMNs occurrence were not analyzed in this study and requires further investigations.

The possible mechanism between HDL and cancer development or progression has not been totally clarified. HDL-associated proteins may have antioxidant, anti-inflammatory, antiangiogenic, and immunomodulatory properties which may contribute to antitumorigenic effects [29]. In addition, cancer cells express high levels of the scavenger receptor type B-I lipoprotein receptor, which promotes lipid internalization and lipoprotein consumption by cancer cells [33]. Low levels of circulating HDL-c may be attributable to the fact that cancer cells utilize a large amount of cholesterol for the formation of new membranes [33]. Moreover, HDL-c levels do not always correlate with HDL function and cholesterol is only one component of HDL [34]. Further studies are required to explore the mechanism involved in the HDL-mediated anti-tumorigenic effects [12]. Longitudinal studies may supply more strong evidence than a cross-sectional study. Clinical trials may be performed to show the role of statins against the malignant transformation of IPMNs.

### Comparisons with other studies and what does the current work add to the existing knowledge

The associations between HDL-c levels and cancer risk have been reported. Associated factors with malignant IPMNs are also been reported. However, few data showed the associations between HDL-c levels and malignant transformation of IPMNs. The current study reported that low HDL-c levels (< 0.7 mmol/L)was associated with malignancy in IPMNs.

## Study strengths and limitations

This study has several limitations. First, the sample size was relatively small, particularly, the small number of malignant IPMNs or carcinoma in BD-IPMNs. Second, it would be better to perform a longitudinal study to show the association between baseline serum HDL-c levels and develop malignant IPMNs. Third, whether low HDL-c level represent a consequential or causal factor in the development of malignant IPMNs need further exploration. Fourth, some confounders were not considered, such as lifestyle and diet habits. Finally, HDL-associated proteins and enzymes were not easily affected by covariates. We did not observe the association between malignant IPMNs and them. Our study is an exploration.

## Conclusions

This study shows that low HDL-c levels are associated with malignant IPMNs and low HDL-c levels may serve as a potential biomarker for identifying malignant IPMNs. Moreover, measurement of HDL-c levels may improve the predictive ability of malignancy in BD-IPMNs. Prospective studies are warranted to investigate the association between HDL-c levels and malignant transformation of IPMNs. HDL-c-raising may be a therapeutic strategy against malignant transformation in IPMNs.

## Data Availability

All data generated or analyzed during this study are included in this published article (and its Supplementary Information files).

## Abbreviations

AUC: area under the curve
BD: branch duct
CA19-9: carbohydrate antigen 19 − 9
CEA: carcinoembryonic antigen
CI: confidence interval
HDL-c: high-density lipoprotein-cholesterol
IPMN: intraductal papillary mucinous neoplasms
LDL: low density lipoprotein
MD: main duct
MPD: main pancreatic duct
MT: mixed-type
OR: odds ratio
ROC: receiver operating characteristic
